# Key points of technical review for the registration of SARS-CoV-2 nucleic acid tests in China

**DOI:** 10.4155/bio-2021-0166

**Published:** 2021-11-15

**Authors:** Hongran Li, Tong Jiao, Peirong Wang, Juanjuan An, Gang Deng, Lei Sun, Jinchun Dong

**Affiliations:** ^1^Center for Medical Device Evaluation, NMPA, 50 Qixiang Road, Haidian District, Beijing, 100081, China

**Keywords:** nucleic acid, registration, SARS-CoV-2, tests

## Abstract

In response to the outbreak of COVID-19, in accordance with the principles of ‘unified command, early involvement, prompt review and scientific approval’ as well as the requirements of ensuring product safety, effectiveness and controllable quality, the Center for Medical Device Evaluation (CMDE) has issued *Key Points of Technical Review for the Registration of SARS-CoV-2 Nucleic Acid Tests* (*Key Points*) to provide the requirements of tests. Because of the sustainability of the pandemic, more efforts and attempts are needed for SARS-CoV-2 detection and control. This article interprets the Key Points issued by the CMDE and provides certain refinements to wider audiences.

A worldwide pandemic caused by a novel coronavirus called severe acute respiratory syndrome coronavirus 2 (SARS-CoV-2) is still not well controlled [1–3]. Globally, there have been over 190 million cases and more than 4 million deaths so far, causing a serious and long-lasting public health emergency [4]. Accurate and rapid detection of SARS-CoV-2 will help patients to access timely treatment, prevent further transmission and ultimately prevent COVID-19 pandemic [5–7]. Real-time fluorescent PCR nucleic acid tests are widely used in the aetiology detection of SARS-CoV-2, with fast, sensitive and specific characteristics. In this paper, SARS-CoV-2 nucleic acid tests are those based on reverse transcription polymerase chain reaction (RT-PCR) to amplify SARS-CoV-2 specific genes and detect the PCR products in real time by fluorescence-labeled probes.

Countries all over the world have their own approval policies for the marketing of SARS-CoV-2 nucleic acid tests. The World Health Organization (WHO) and US Food and Drug Administration (FDA) initiated their emergency procedure, issued and updated related guidance about Emergency Use Listing (EUL, for WHO)/Emergency Use Authorization (EUA, for FDA) of SARS-CoV-2 nucleic acid tests at different times. SARS-CoV-2 tests are managed as Class III medical devices in China. If local or abroad manufacturers seek to develop and distribute nucleic acid tests in China, they should register and apply for the approval in accordance with the requirements of the National Medical Products Administration (NMPA) and the application materials should be reviewed at the Center for Medical Device Evaluation (CMDE). The CMDE drafted the *Key Points of Technical Review for the Registration of SARS-CoV-2 Nucleic Acid Tests* (Key Points) on 20 January 2020, and issued it on 12 February 2020 [8] to provide guidance for reviewers and commercial manufacturers.

Based on the requirements of the Key Points [8], more than 30 SARS-CoV-2 nucleic acid tests have been approved to be marketed in China, and the products of many manufacturers are in the application process or the development phase before application.

The Key Points aims to help manufacturers to prepare and formulate application dossiers for the registration of SARS-CoV-2 nucleic acid tests in China and to provide reference for technical review departments in reviewing the application data [8]. The Key Points is the general requirement for SARS-CoV-2 nucleic acid tests, and applicants should determine whether the content is applicable according to the specific characteristics of products [8]. If the content is not applicable, the reasons and corresponding scientific basis should be specified and the content of registration application data should be enriched and refined according to the specific characteristics of products.

This article interprets the Key Points issued by the CMDE to facilitate understanding for wider audiences. This article also provides certain refinements, such as the requirements of variants, the limit of detection and inclusivity, based on continuous understanding of SARS-CoV-2 and the practice of technical reviewers. The authors believe a sufficient and accurate understanding of the requirements will improve the design, development and application for marketing of SARS-CoV-2 nucleic acid tests.

## Scope of application

After the outbreak of the pandemic, WHO, FDA and CMDE issued respective marketing requirements for SARS-CoV-2 tests [9,10]. There are certain differences in the requirements of various institutions. China requires more sufficient performance evaluation and more research data such as raw materials and control materials. At present, more than 30 SARS-CoV-2 nucleic acid tests have been approved in China, all of which are produced by Chinese manufacturers. Based on the Key Points released by the CMDE [8], this paper has refined and updated some contents, such as adding the requirements of viral variants detection. This paper is helpful for both local and abroad manufacturers to understand the current review requirements of the CMDE for SARS-CoV-2 tests and for the registration and application of their products in China.

SARS-CoV-2 nucleic acid tests are used for *in vitro* qualitative detection of SARS-CoV-2 nucleic acid in samples such as throat swabs, nasopharyngeal swabs, bronchoalveolar lavage fluid, sputum, aspirated liquid and other respiratory secretions [11].

## Performance evaluation

Manufacturers should submit the research data of all performance evaluation for the tests manufactured under the production environment conforming to quality management system of manufacturers, including detailed data: specific research methods, experimental scheme, experimental data, statistical analysis and so on. The basic information of samples used for performance evaluation also needs to be clarified, such as sample source, sample type, collection and processing method, dilution method and value determination process. The samples for performance evaluation generally should be real samples. If dilution is involved, a negative matrix consistent with the applicable sample type should be used. The following performances are required to be validated.

### Extraction & purification performance of RNA

Before the detection of target RNA, the extraction and purification step is necessary [12]. The purpose of the step is not only to extract the maximum amount of target RNA but also to have the corresponding purification function and to remove PCR inhibitors as much as possible. No matter whether detection tests contain the components of RNA separation and purification, manufacturers should fully validate the extraction efficiency and purity of RNA and provide detailed validation data. Generally speaking, SARS-CoV-2 nucleic acid tests contain independent RNA extraction steps, but there are also some fully integrated tests (all steps of sample processing, amplification and detection combined in a single, disposable device), in which it is not possible to evaluate extraction efficiency or RNA purity. For these tests, the original clinical samples and their extracted nucleic acids are tested respectively to compare the consistency of the results, in order to prove that the extraction performance of the test meets the requirements.

### Limit of detection

The real clinical samples containing SARS-CoV-2 or pseudovirions RNA segments are diluted gradiently in appropriate substrate (being consistent with applicable samples). Each concentration gradient should be detected with at least three replicates. Taking the lowest concentration level that can be detected by 100% as the estimated LOD, samples with several gradient concentrations are prepared around the estimated LOD. Each concentration should be detected at least 20-times. The lowest concentration level with a positive detection rate of ≥95% should be taken as the established LOD [13]. Scientific methods should be used to determine the virus quantity value in the sample and the value should be consistent with the value of sensitivity reference in the national reference of the National Institutes for Food and Drug Control.

In the establishment of the LOD, it is required that manufacturers use at least five representative SARS-CoV-2 samples in gradient dilution from independent individuals with different sample collection time and regions.

At least another 5 virus samples from independent individuals with different sample collection time and regions are selected to validate the established LOD, and the positive detection rate should reach ≥95%. Detailed determination method and validation results of virus quantity value are required.

LOD is a key performance characteristic to ensure that positive samples can be detected when the virus concentration of SARS-CoV-2 is low. Manufacturers should reasonably design the product and optimize the reaction system to ensure LOD meet the requirements.

According to *Guidelines for the Diagnosis and Treatment of Coronavirus Disease 2019* [14], highly sensitive reagent is recommended (LOD ≤500 copies/ml). The National Health Commission of the People's Republic of China released *Notice on Further Strengthening SARS-CoV-2 Nucleic Acid Detection in Fever Clinics* on 1 September 2021. According to this notice, fever clinics should be equipped with approved nucleic acid detection equipment with suitable flux and select a SARS-CoV-2 nucleic acid test with stable performance and high sensitivity (LOD ≤500 copies/ml) to ensure the sensitivity of nucleic acid detection.

### Inclusivity

Manufacturers are required to validate at least 20 virus samples (inactivated positive clinical samples or isolated cultures) from different sources of different sample collection time and regions. It is recommended to select epidemic strains in China and global representative strains. When these samples or strains are diluted to LOD level, the positive detection rate should be ≥95%. Samples or strains should be tested many times to evaluate the repeatability. In the inclusive study, artificial samples such as plasmids or pseudoviruses cannot be used.

### Analytical specificity

#### Cross-reactivity

The sequence homology of SARS-CoV-2 and other human coronaviruses makes them most likely cross-reactive with each other [15–17]. Individuals infected with influenza A and B may have similar symptoms as those infected with SARS-CoV-2, and coinfection of influenza [18,19] or other common respiratory pathogens [20–22] has been reported. Many respiratory pathogens related guidances [23] suggested several cross-reactivity pathogens. Validation of cross-reactivity for SARS-CoV-2 tests should include the following substances.

(1) Endemic human coronavirus (HKU1, OC43, NL63 and 229E), SARS coronavirus and MERS coronavirus; H1N1 (2009 novel influenza A H1N1 virus, seasonal H1N1 influenza virus), H3N2, H5N1, H7N9, influenza B Yamagata and Victoria, respiratory syncytial virus A and B, parainfluenza virus 1, 2 and 3, rhinovirus group A, B and C, adenovirus 1, 2, 3, 4, 5, 7 and 55, enterovirus group A, B, C and D, human metapneumovirus, EB virus, measles virus, human cytomegalovirus, rotavirus, norovirus, mumps virus and varicella-zoster virus; *Mycoplasma pneumonia* and *Chlamydia pneumoniae*; *Legionella*, *Bordetella pertussis*, *Haemophilus influenza*, *Staphylococcus aureus*, *Streptococcus pneumonia*, *Pyogenic streptococcus*, *Klebsiella pneumonia* and *Mycobacterium tuberculosis*;
  *Aspergillus fumigates*, *Candida albicans*, *Candida glabrata*, *Cryptococcus neoformans*, and so on.
  It is suggested that manufacturers validate cross-reactivity at the medical decision levels of viral and bacterial infection. Generally, the concentration level of bacterial and viral infection are 10^6^ cfu/ml and 10^5^ pfu/ml or higher, respectively. Manufacturers are required to provide test data on the validation of cross-reactivity, such as the sources, species or types and concentration confirmation of the virus and bacteria.
(2) Human genome DNA

#### Interference of endogenous & exogenous substances

The interference of potential endogenous and exogenous substances should be evaluated. Recommended substances can be referred to the issued Key Points [8]. The potential highest concentration of interfering substances, which is ‘worst case’ condition, should be evaluated using samples at the critical positive level of SARS-CoV-2 virus. Statistical analysis of Ct value is required for the results of interference research. Results expressed only as negative and positive are inadequate.

### Evaluation of the detection ability of SARS-CoV-2 variants

Since the outbreak of novel coronavirus pneumonia, there have been many variants worldwide [24–26], such as the British b.1.1.7 variant [27], the South African b.1.351 variant [28,29], the Brazilian P.1 variant, the Brazilian n.9 variant, the Finnish fin-796h variant, the Nigerian b.1.525 variant, the French B.1 variant, the Indian b.1.617 double variant (including b.1.617.1, b.1.617.2 and b.1.617.3) [30–34] and the Columbian Mu variant (B1.621 and B1.621.1). Some of the variant strains may result in enhanced transmission capacity [35,36].

If there is a mutation in the detection area, especially a change in primer/probe binding site, the detection performance of the reagent may be affected, so it is necessary to evaluate different variants, including the Alpha, Beta, Gamma, N.9, P.2, Fin-796H, Eta, B.1, B.1.616, Delta, Kappa, Iota, Lambda, Epsilon and Mu variants.

The manufacturer should timely supplement the evaluation of other variants according to the virus mutation. In the evaluation process, *in silico* analysis should be carried out to compare sequences of the detection area of the reagent and primer/probe. Results could be a report including the sequence number of the variants, the name of the amplified gene, the mutation site located at the specific position of primer/probe, the mutation rate and the comparison diagram between the target amplification area and the variant (with the primer and probe in the figure).

If bioinformatics analysis shows that the detection area contains mutation sites, it should be considered whether the mutation has a potential impact on the detection ability of the reagent. The sites with possible influence should be listed and validated by synthetic samples or real clinical specimens (if available). The validation includes LOD and precision of all applicable sample types. If reagents could not detect variant strains, it is advised that the product design be modified and the performance of the product be re-evaluated.

### Precision

Manufacturers should make reasonable requirements for the evaluation criteria of precision, including standard deviation or coefficient of variation (CV). Since artificial samples cannot reflect all possible variance factors of clinical samples, precision evaluation should use several clinical samples and precision evaluation tests should contain the separation and purification steps of nucleic acid. The precision evaluation of such products mainly include the following requirements.

Multiple factors, such as detection tests (including nucleic acid separation and purification components), device, operator, site, detection run and other elements should be considered for the precision analysis. Manufacturers can refer to EP5 for 20-day within-laboratory precision evaluation [37]. In addition, an inter-lot precision study is required.

The clinical samples used for precision evaluation should include at least 3 levels, including negative samples, critical positive samples and moderate/strong positive samples. The appropriate precision acceptance criteria should be set in accordance with product characteristics. Each detection of clinical samples should be finished from the extraction of RNA.

For negative samples whose concentration are lower than LOD or equal to zero concentration, the negative detection rate should be 100% (n ≥ 20). For critical positive samples whose concentration are slightly higher than LOD of the kit (about 1.5× LOD), the positive detection rate should be ≥95% (n ≥ 20). For moderate/strong positive samples whose concentration are higher than 5× LOD/10× LOD, the positive detection rate should be 100% and CV ≤10% (n ≥ 20).

### Validation of manufacturer reference materials

Manufacturer reference materials should be tested using three lots of products and detailed experimental data should be provided, in accordance with the setting of manufacturer reference materials in the research data of main raw materials.

### Other problems to be noted

For a product applicable to several instrument models, the performance evaluation data for all models of instruments listed in the product Instruction for Use (IFU) should be provided. For each sample type applicable to the product, the manufacturer should evaluate the performance, respectively. Key requirements for test performance evaluation are summarized in Table 1.

**Table 1.** Key requirements for test performance evaluation.

Performance characteristics	Key requirements
Extraction and purification of nucleic acid	The extraction efficiency and purity of RNA should be fully validated.
Limit of detection	Establishment and validation of limit of detection should be carried out with 5 representative SARS-CoV-2 clinical samples, respectively
Inclusivity	At least 20 different clinical samples should be used to validate limit of detection and repeatability
Analytical specificity	Cross-reactivity as well as endogenous and exogenous interfering substance analysis should be included
Evaluation of the detectionability of SARS-CoV-2 variants	Bioinformatic analysis should be carried out to compare sequences for the detection area of the reagent and primer/probe. If bioinformatic analysis shows that the detection area contains mutation sites, it should be considered whether the mutation has a potential impact on the detection ability of the reagent. The validation includes LOD and precision of all applicable sample types.
Precision	Negative samples, critical positive samples and moderate/strong positive samples should be evaluated
Manufacturer reference materials	Detecting manufacturer reference materials using three lots of products
Validation of models of instrument	The performance on all models of instruments listed in the product IFU should be evaluated
Validation of different sample types	The performance for each sample type applicable to the product should be evaluated, respectively

## Registered testing

The National Institutes for Food and Drug Control has developed national reference for SARS-CoV-2 nucleic acid tests. According to the *Regulation of Registration and Filing for in Vitro Diagnostic Tests* (no. 48) [38], if there is an applicable national reference, the reagent should be tested with the national reference. As Class III *in vitro* diagnostic reagents, three lots of SARS-CoV-2 nucleic acid tests are needed in registered testing.

## Determination of positive cut-off value

Determination of positive cut-off value mainly refers to the determination of Ct value of virus nucleic acid detection, namely the critical value of result judgment. The diversity and representativeness of sample source should be considered, such as different ages, genders, geographic regions, infection stages and other factors, in the research of positive cut-off value. Samples with different concentrations of negative, positive and weak positive should be included. The positive cut-off value (Ct value) of each target should be studied by receiver operating characteristic (ROC) curve, and the interpretation rules of double positive and single positive results of each target should be studied. If there is a gray area of cut-off value, manufacturers are required to provide the determination data of it.

## Research data on main raw materials

The main raw materials of SARS-CoV-2 nucleic acid tests include primers, probes, enzymes, deoxynucleoside triphosphate (dNTP), control materials, separation and purification components of nucleic acid (if any) and reference materials. Manufacturers are required to provide the research data on the material selection (such as comparative screening test), the source, the preparation process and the quality inspection data of this raw material. For control materials, the study data of target value should also be provided. If main raw materials are made by the manufacturer itself, it is required to provide the detailed preparation process. If main raw materials are purchased from other supplier, the supplier name, the quality standard and certification of analysis of main raw materials from supplier should be provided.

### Primer & probe

Primers and probes are important raw materials for determining whether the tests can accurately and specifically detect SARS-CoV-2. At present, primers designed for SARS-CoV-2 mainly focus on open reading frame 1ab (ORF1ab) and nucleocapsid protein (N) genes; primers for E gene may also be added. At present, there are dual-target or triple-target designs in products approved in China. Manufacturers are required to describe the target sequence selection and design principles of the primer and probe and provide the genetic loci of the RNA sequence and target sequence of the primer and probe as well as their corresponding situation. It is suggested that two or more sets of primers and probes be designed for each virus for screening. Sequence alignment with public SARS-CoV-2 sequences (including variant strains) and alignment with other pathogens that are prone to cross-reaction as well as wet testing should be used to evaluate the inclusivity and specificity (e.g. cross-reactivity) of the primer and probe to select the optimal combination. The quality standard for the primer and probe should include at least sequence accuracy, purity, concentration and functional experiment.

### Deoxynucleoside triphosphate

Deoxynucleoside triphosphate (dNTP) includes 2'-deoxyadenosine 5'-triphosphate (dATP), deoxyuridine triphosphate (dUTP), 2'-deoxyguanine 5'-triphosphate (dGTP), 2'-deoxycytidine 5'-triphosphate (dCTP), thymidine 5'-triphosphate (dTTP). Manufacturers are required to provide the detailed validation data for its purity, concentration and function.

### Enzymes

The required enzymes mainly include DNA polymerase, reverse transcriptase and uracil-DNA glycosylase. Manufacturers are required to evaluate and validate enzymatic activity and function, respectively.

### Control materials in kit

The control materials provided with the test kit should participate in the whole process of sample processing as well as the parallel extraction and detection of RNA in order to conduct reasonable quality control of the whole process, including nucleic acid extraction, reverse transcription and PCR amplification. The application documents should contain the detailed description and testing data about controls, such as selection of raw materials, preparation, valuing process and concentration range. It should be clarified in the IFU that control materials participate in the nucleic acid extraction process and provide the range of the test results (e.g., Ct value) of controls. Controls that will be provided with the test kit include the following.

#### Negative control

Negative control should not contain the target sequence detected by the kit.

#### Positive control

Positive control should contain natural or synthetic target nucleic acid sequences that can be detected by the kit; pseudovirus is generally used for marketed products. Plasmid is not recommended, because plasmid cannot monitor the extraction and reverse transcription process.

#### Internal control

Internal control can be used to conduct quality control for the false negative result caused by in-pipe inhibition. Applicants should make precise validation for the design of the internal control primer, probe and template concentration, ensuring the obvious positive curve shown by internal fluorescent channel and minimizing the inhibition caused by target gene detection. The marketed tests are often labeled with endogenous internal control (using gene RNaseP/β-globin and so on) and exogenous internal control (such as RNA pseudovirus); there are also exogenous added sequences.

It is suggested that manufacturers scientifically set internal control and monitor the quality of the sample to be tested and the reaction system of tests.

### Manufacturer reference materials

Manufacturer reference materials are a panel of samples established by the manufacturer for semi-finished product inspection, delivery inspection and other daily tests after production, to ensure that each lot of products maintains good performance.

Manufacturer reference materials should be set according to the actual demands for the validation of product performance, including positive reference materials, negative reference materials, LOD reference materials and repeatability reference materials. Manufacturer reference materials usually need to be prepared with clinical samples. Only SARS and MERS coronavirus, if it is difficult to obtain samples or virus strains, can use pseudovirus.

Positive reference materials should include at least 5 samples of different sources and concentration.

Negative reference materials mainly involve the validation of cross-reactivity, which should include coronavirus (HKU1, OC43, NL63 and 229E), SARS coronavirus, MERS coronavirus, influenza virus, parainfluenza virus, respiratory syncytial virus, adenovirus, and so on.

The concentration of LOD reference materials can be ≥95% positive detection level or slightly higher, such as 100% positive detection level. Repeatability reference materials are recommended to include the samples at high and low concentrations (around LOD).

## Research data on main production process & reaction system

The main production process of the product can be presented in the form of a flow chart, and the determination basis for the main production process should be specified.

Considering the need for SARS-CoV-2 laboratory testing, applying products should generally detect upper respiratory tract samples (oropharyngeal swabs, nasopharyngeal swabs) and lower respiratory tract samples (sputum and bronchoalveolar lavage fluid).

The method for sample collection and processing should be described in details. Each applicable collection and processing method should be validated and specified in the instructions.

 For the time point of sample collection, the manufacturer should consider the following aspects: whether it is affected by the course of disease, clinical symptoms, medications and other factors. It is also required to clarify the materials of sampling swab head and swab rod, the compositions and volume of preservation solution/sampling solution and the sample container.

The digestive fluid for post-treatment of sputum collection, which may contain dithiothreitol or protease K, acetylcysteine should be optimized.

For the biosafety, inactivation procedure of sample is generally included in the sample preparation protocol. These inactivation method, such as thermal inactivation or chemical inactivation, should be validated. Thermal inactivation is generally carried out by heating at 56°C for 30 min. Chemical inactivation can be carried out by directly putting the virus into the virus lysate containing guanidine isothiocyanate or guanidine hydrochloride after collection to inactivate the virus quickly. It is necessary to study concentration, lysate dosage, sample dosage and sample storage time after adding lysate.

Research data for confirming the optimum reaction system include sample amount, concentrations of various enzymes, primer/probe concentration, dNTP concentration and positive ion concentration, as well as the temperature, time and cycle number of all stages of reaction. It is suggested that manufacturers expand the total reaction system and sample loading amount under the circumstance of ensuring the extraction purity of nucleic acid, to improve product sensitivity.

In case of any differences in the reaction conditions of different applicable instrument models, they should be described in detail and the validation data should be submitted.

If the declared product contains nucleic acid separation or purification tests, the manufacturer must submit the research data on process optimization for the separation or purification process of nucleic acid.

## Evaluation of stability

Study on stability mainly consists of two parts, the stability of tests and the stability of applicable samples.

### Stability of tests

The stability of tests mainly includes the shelf-life stability, in-use stability, accelerated stability under damage of high temperature and transport simulation, and limit of times of freezing and thawing. Research data on stability should include the determination basis of the research method, the specific implementation plan, detailed research data and conclusions [39]. For shelf-life stability study, at least three lots of reagents in final customer package are required to be evaluated and the products should be stored under the recommended conditions from manufacture time to the expiry date.

### Stability of applicable samples

Considering the highly degradable feature of virus RNA, manufacturers should also study stability of applicable samples stored under storage conditions (such as at refrigeration temperature or under frozen condition). The untreated samples after collection, samples in lysate or preservation solution and samples inactivated should be analyzed separately. Since the interfering substances in different sample types may be distinct and their impacts on the stability of virus RNA may be different, each of sample types should be studied if a product is applicable to more than one sample types (such as swab, sputum and lavage fluid).

 For samples stored under frozen condition, the number of freezing and thawing times should also be evaluated. If the nucleic acid extract cannot be detected immediately, the storage conditions and stability of the extracted nucleic acid need to be studied as well.

## Clinical evidence

The clinical trial for SARS-CoV-2 nucleic acid tests should be conducted in at least 3 clinical trial institutions (including centers for disease control and prevention if needed). In clinical trial, similar marketed products should be selected as comparator. Meanwhile, the detection results of the candidate tests are considered to be compared with the clinical diagnosis/exclusion results [40]. In accordance with the *Diagnosis Scheme of Novel Coronavirus Pneumonia, Confirmation Procedures for the First Case of Novel Coronavirus Pneumonia in All Provinces (Regions and Cities) of China* and other documents issued by the National Health Commission of the People's Republic of China, case confirmation methods should be formulated according to the confirmation procedures for the first case of all provinces and those of the second and following cases of all provinces specified in the above documents. It is not recommended to use the results of a single tests in a trial site as confirmation results.

The clinical trial aims to validate the equivalence between the candidate test and a similar product in clinical application as well as clinical reference standard using clinical specimens. Statistical analysis is generally conducted to summarize the results in the form of a 2 × 2 table, then to calculate the coincidence rate, clinical sensitivity, clinical specificity and confidence interval of the candidate test and the comparator/reference accordingly. Due to differences in product methodology and other characteristics, the clinical trial results should be analyzed case by case.

## Conclusion

The Key Points for the SARS-CoV-2 tests was issued to facilitate the availability of SARS-CoV-2 nucleic acid tests to response to the COVID-19 public health emergency effectively [8]. It describes the requirements for SARS-CoV-2 nucleic acid tests for marketing during the COVID-19 pandemic in China. This article is an attempt to expand clarification of the Key Points [8] to wider audiences based on the current understanding of SARS-CoV-2 to facilitate the development and application of SARS-CoV-2 nucleic acid tests. Since the regulatory requirements for SARS-CoV-2 products are different among regulatory agents, a correct and comprehensive understanding of each guidance will improve the design, development and application for marketing or emergency use during this pandemic. With the uncontrolled spread and evolution of SARS-CoV-2 in the world, continuous prevention and countermeasures are necessary, and a comprehensive explanation of the requirement for SARS-CoV-2 tests is needed for wider audiences. With the deeper understanding of SARS-CoV-2 and the development of the COVID-19 pandemic, the Key Points will be updated accordingly.

## Future perspective

Rapid and accurate laboratory testing of SARS-CoV-2 is essential for early discovery, early reporting, early quarantine, early treatment and prevention of epidemic transmission. In the diagnosis of COVID-19, positive results of viral nucleic acid tests as direct etiological evidence are regarded as one of the key indicators. In the past few months of clinical practice and research, SARS-CoV-2 nucleic acid detection technology has proved to have a good positive detection rate, especially in the first two weeks during disease, and can effectively diagnose related viral infections. However, the false negatives of nucleic acid tests are still frequently reported, and the main reasons include patient sampling quality, viral load and distribution, viral mutation and the standardization of the detection process. It is necessary to reasonably regulate the production and registration of SARS-CoV-2 tests with consistent and scientific standards.

**Executive summary**

**Background**

- The purpose of this article is to guide manufacturers to prepare and formulate application dossiers for the registration of SARS-CoV-2 nucleic acid tests and to provide reference for technical review departments to review the application data.

**Scope of application**

- SARS-CoV-2 nucleic acid tests are used for the *in vitro* qualitative detection of SARS-CoV-2 nucleic acid in samples such as throat swab, nasopharyngeal swab, bronchoalveolar lavage fluid, sputum, aspirated liquid and other respiratory secretions.

**Performance evaluation**

- All analytical performance of candidate tests should be evaluated and validated using reagents manufactured under the control of quality management system.

**Extraction & purification performance of RNA**

- Manufacturers should fully validate the extraction efficiency and purity of RNA and provide detailed validation data.

**Limit of detection**

- Establishment and validation of limit of detection should be carried out with at least five representative SARS-CoV-2 samples from different sources.

**Inclusivity**

- Manufacturers are required to validate 20 virus samples from different sources of different sample collection time and regions.

**Analytical specificity**

- Cross-reactivity should be evaluated for medical decision levels of virus and bacterial infection and human genome DNA. The interference of potential endogenous and exogenous substances should be evaluated.

**Evaluation of the detection ability of SARS-CoV-2 variants**

- Bioinformatic analysis should be carried out to compare sequences for the detection area of the reagent and primer/probe. If bioinformatic analysis shows that the detection area contains mutation sites, it should be considered whether the mutation has a potential impact on the detection ability of the reagent. The validation includes LOD and precision of all applicable sample types.

**Precision**

- Many factors and a reasonable evaluation period should be considered for precision evaluation. Negative samples, positive samples and moderate/strong positive clinical samples should be evaluated.

**Validation of manufacturer reference materials**

- Manufacturer reference materials should be tested using three lots of products.

**Other problems to be noted**

- For the product applicable to several instrument models, performance evaluation data for all models of instruments listed in the product IFU should be provided. For each sample type applicable to the product, manufacturers should evaluate the performance respectively.

**Registered testing**

-  Three lots of SARS-CoV-2 nucleic acid tests are needed in registered testing using national reference.

**Determination of positive cut-off value**

- The diversity and representativeness of sample source should be considered and the analysis of ROC curve is recommended.

**Research data on main raw materials**

- Manufacturers are required to provide research data on the selection, source, preparation and quality standard of main raw materials.

**Primer & probe**

- Manufacturers are required to elaborate the design principles of the primer/probe and provide the genetic loci of RNA sequence and target sequence of the primer/probe as well as their corresponding situation.

**Deoxynucleoside triphosphate**

- Manufacturers are required to provide detailed validation data for its purity, concentration and function.

**Enzymes**

- Manufacturers are required to evaluate and validate enzymatic activity and function respectively.

**Control materials in kit**

- Control materials in kit include negative controls, positive controls and internal controls. The application materials should contain a detailed description of the testing data on kit control materials, such as selection of raw materials, preparation, valuing process and concentration range.

**Manufacturer reference materials**

- The internal reference materials of manufacturers should be set according to the actual demands for the validation of product performance, including positive reference materials, negative reference materials, reference materials of LOD and repeatability reference materials.

**Research data on main production process & reaction system**

- For confirmation of the main production process and reaction system, several factors of all stages of reaction should be optimized.

**Evaluation of stability**

- Stability of tests and stability of applicable samples should be evaluated.

**Clinical evidence**

- The clinical trial for the SARS-CoV-2 nucleic acid tests should be conducted in at least three clinical trial institutions.

**Conclusion**

- The Key Points for SARS-CoV-2 tests was issued to facilitate the availability of SARS-CoV-2 nucleic acid tests to respond to the COVID-19 public health emergency effectively.
